# A carbon monoxide releasing metal organic framework nanoplatform for synergistic treatment of triple-negative breast tumors

**DOI:** 10.1186/s12951-022-01704-2

**Published:** 2022-11-24

**Authors:** Yiyang Cong, Bo Sun, Jianlun Hu, Xiaoyang Li, Yanan Wang, Jingyi Zhang, Dongzhi Yang, Weifei Lu, Zhi Ding, Xiaofeng Wang, Hao Hong

**Affiliations:** 1 State Key Laboratory of Pharmaceutical Biotechnology School of Life Sciences, Nanjing University, 163 Xianlin Avenue, Nanjing, 210093 China; 2 Jiangsu Key Laboratory of New Drug Research and Clinical Pharmacy, Xuzhou Medical University, Xuzhou, 221004 Jiangsu China; 3 State Key Laboratory of Pharmaceutical Biotechnology, Chemistry and Biomedicine Innovation Center (ChemBIC), Jiangsu Key Laboratory of Molecular Medicine, Medical School of Nanjing University, Nanjing, 210093 China; 4 College of Veterinary Medicine, Henan Agriculture University, Zhengzhou, 450002 Henan China; 5 Nanjing Key Laboratory of Advanced Functional Materials, Nanjing Xiaozhuang University, Nanjing, 211171 People’s Republic of China

**Keywords:** Metal–organic framework, Catalysis, Carbon monoxide, Imaging, Synergistic therapy

## Abstract

**Background:**

Carbon monoxide (CO) is an important signaling molecule participating in multiple biological functions. Previous studies have confirmed the valuable roles of CO in cancer therapies. If the CO concentration and distribution can be controlled in tumors, new cancer therapeutic strategy may be developed to benefit the patient survival.

**Results:**

In this study, a UiO-67 type metal–organic framework (MOF) nanoplatform was produced with cobalt and ruthenium ions incorporated into its structure (Co/Ru-UiO-67). Co/Ru-UiO-67 had a size range of 70–90 nm and maintained the porous structure, with cobalt and ruthenium distributed uniformly inside. Co/Ru-UiO-67 was able to catalyze carbon dioxide into CO upon light irradiation in an efficient manner with a catalysis speed of 5.6 nmol/min per 1 mg Co/Ru-UiO-67. Due to abnormal metabolic properties of tumor cells, tumor microenvironment usually contains abundant amount of CO_2_. Co/Ru-UiO-67 can transform tumor CO_2_ into CO at both cellular level and living tissues, which consequently interacts with relevant signaling pathways (e.g. Notch-1, MMPs etc.) to adjust tumor microenvironment. With proper PEGylation (pyrene-polyacrylic acid-polyethylene glycol, Py-PAA-PEG) and attachment of a tumor-homing peptide (F3), functionalized Co/Ru-UiO-67 could accumulate strongly in triple-negative MDA-MB-231 breast tumors, witnessed by positron emission tomography (PET) imaging after the addition of radioactive zirconium-89 (^89^Zr) into Co-UiO-67. When applied in vivo, Co/Ru-UiO-67 could alter the local hypoxic condition of MDA-MB-231 tumors, and work synergistically with tirapazamine (TPZ).

**Conclusion:**

This nanoscale UiO-67 MOF platform can further our understanding of CO functions while produce CO in a controllable manner during cancer therapeutic administration.

**Graphical Abstract:**

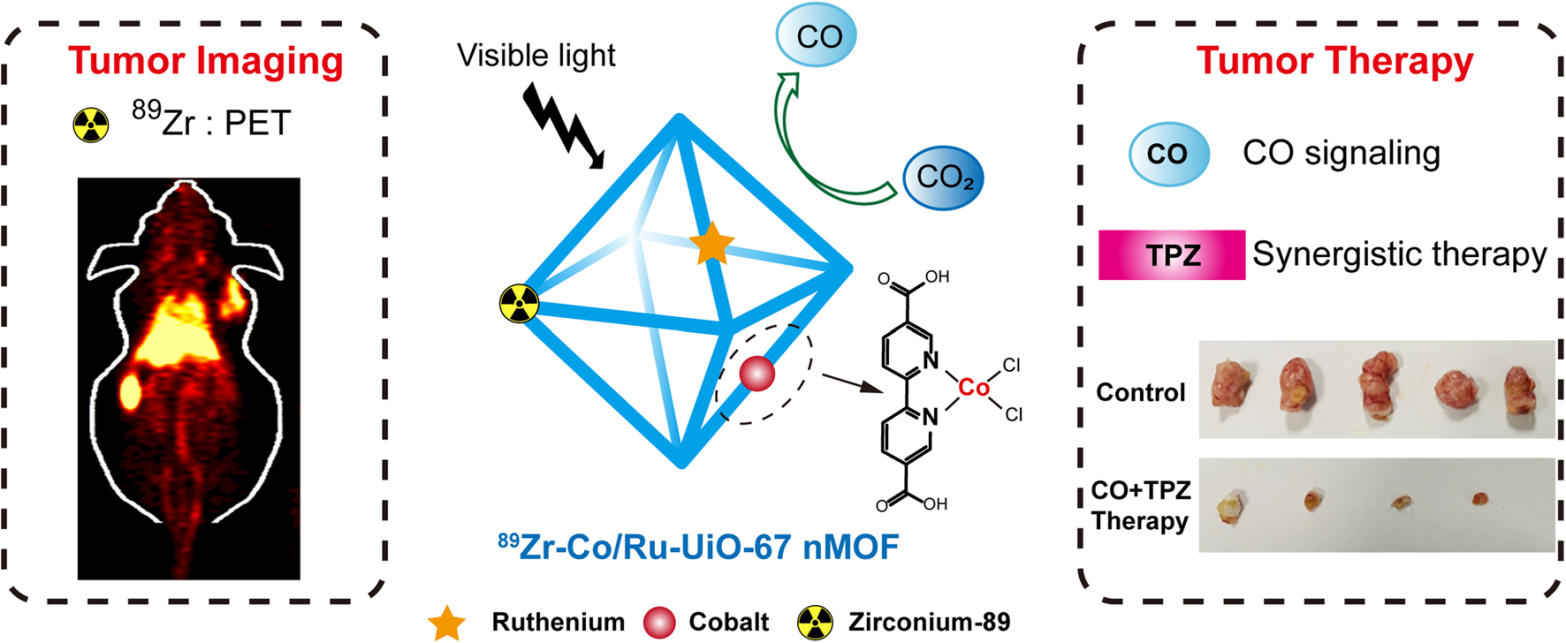

**Supplementary Information:**

The online version contains supplementary material available at 10.1186/s12951-022-01704-2.

## Introduction

As a gaseous signaling molecule, carbon monoxide (CO) is actively involved in various physiological or pathological processes [1–3]. With continuous exploring research, scientific community now revealed that CO was far beyond a well-known toxic gas. It was currently considered as a “gasotransmitter”, and previous studies confirmed that CO could interact with mitochondria and interfere with respiration chains, resulting in concentration changes of reactive oxygen species (ROS) [4]. CO was used as a therapeutic agent for many diseases, such as inflammation [5], cardiovascular disease [6], cancer [7], among many others [8]. To fully utilize the therapeutic power of CO, how to maintain its production in a controllable manner in vivo is quite important. Thus, a selective delivering vector for CO can be beneficial.

Metal organic framework (MOF) materials, which are composed from metal ion clusters and organic bridging ligands, can be generated from different methods. Nanoscale MOF (nMOF) possessed porous structure, and combined the advantages of metal ions and the organic ligands, which were frequently used for catalysis [9], sensing [10], imaging [11], and other biomedical applications [12]. nMOF can accommodate a variety of cargos, including CO, its precursors, and other gaseous biomolecules [13, 14]. Specifically, in one recent study, rhenium or cobalt doped UiO-67 nMOF could efficiently transfer CO_2_ to CO upon light irradiation [15]. From the unique properties of nMOFs, such a nanoplatform to control local CO concentration may be readily designable.

Tirapazamine (TPZ), which belongs to the category of hypoxia-activated prodrugs (HAPs) [16], was selected as the therapeutic cargo in this study. As an aromatic N-oxide, TPZ can be reduced under a hypoxia condition to generate a radical species, resulting in the DNA damages or protein damages triggered by various radicals (e.g. hydroxyl or benzotriazinyl) [17]. TPZ was currently used in the clinic for the treatment of hypoxic solid tumors [18]. Previously, TPZ was combined with a CO-releasing nanoplatform to produce better treatment efficacy [19]. Thus, a synergistic therapeutic effect between TPZ and CO could be anticipated when they can be delivered selectively in the tumor area. However, an optimal dose for TPZ and CO for more efficient therapy should be explored.

**Fig. 1:**
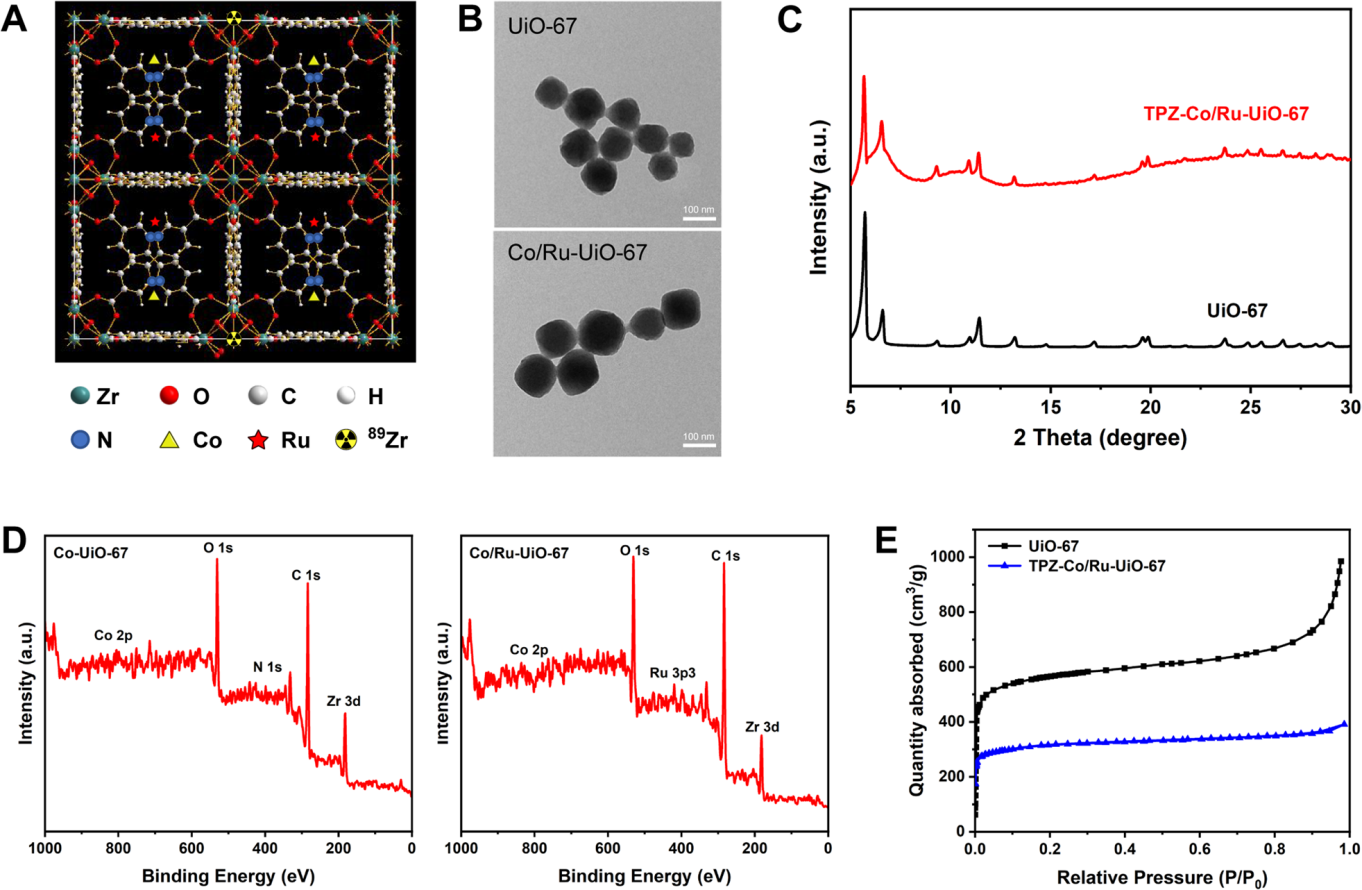
The synthesis and material characterization of UiO-67 conjugates. **A** Schematic illustration of Co/Ru-UiO-67 space structures. The elemental distribution was also shown for demonstration purpose (some elements such as Co, Ru, and ^89^Zr was not quantitative to a real structure). **B** TEM images of UiO-67 and Co/Ru-UiO-67. **C** XRD spectra of UiO-67 and TPZ-Co/Ru-UiO-67@Py-PAA-PEG. **D** XPS measurements to reveal the core elements on Co-UiO-67 and Co/Ru-UiO-67. **E** Brunauer–Emmett–Teller (BET) measurement of UiO-67 as well as TPZ, cobalt, and ruthenium loaded UiO-67 to determine their surface areas

Our goal in this study is to develop a nMOF platform to catalyze carbon dioxide to CO in vivo. Inspired by the previous findings, we hope to build an in situ CO “generator”, which can not only impose selective cancer cell killing, but also be traceable by different imaging techniques. UiO-67 type nMOF, which is here composed of zirconium clusters and 2,2-bipyridine-4,4′-dicarboxylic acid, was used as the core material here. Based on multiple previous reports [20–22], UiO-67 MOF had an octahedron crystal structure, and the existence of 2,2-bipyridine inside UiO-67 could be used for the coordination of various metal cations. Cobalt was coordinated inside UiO-67 structure (forming Co-UiO-67) for catalysis of CO_2_, since its catalytic energy barrier was relatively low (0.86 eV) [15]. Moreover, different research groups confirmed that UiO-67 type nMOF was biocompatible and readily useful for cancer theranostics [23, 24]. This nMOF platform can consume the abundant CO_2_ in the tumor microenvironment (TME) and convert it to CO, which can partially alter the TME. To improve the whole light catalysis sensitivity, ruthenium (II) was also loaded inside the UiO-67 structure to serve as a photosensitizer [23]. The cobalt and ruthenium-loaded UiO-67 was named Co/Ru-UiO-67 in this study (Fig. 1A and TOC figure).

Nucleolin in nucleus regulates the synthesis of rRNA and biogenesis of ribosome. However, nucleolin translocation from nucleus to cell membrane is considered as a tumorigenic event. It was reported that multiple types of cancer cells and activated (angiogenic) endothelial cells demonstrated the overexpression of nucleolin on their membrane surface [25, 26]. Thus, nucleolin could serve as a potential tumor cell target for delivery of cancer theranostic agents. In order to enhance the tumor-targeting efficacy, a F3 peptide against nucleolin was attached to the surface of Co-UiO-67, since previously it was labeled with isotopes such as ^111^In or ^225^Ac for theranostic utilization [27, 28]. Consistent with our previously adopted strategy, a pyrene-derived polyethylene glycol (PEG) was conjugated on Co-UiO-67 nMOF via π-π interaction [29]. A positron-emitting isotope, zirconium-89 (^89^Zr) [30], was embedded in Co-UiO-67 to trace the metabolic behavior of the material in vivo by positron emission tomography (PET) imaging [31]. After the determination of its tumor-homing properties and pharmacokinetic behavior, Co/Ru-UiO-67 was administered intravenously, while light irradiation was conducted at the time points where the tumor uptake of the material reached the peak. With the sustainable production of CO upon light irradiation, Co/Ru-UiO-67 platform may serve as a new way for combinational cancer therapy.

## Results and discussions

### Fabrication and characterization of CO-catalysis Co/Ru-UiO-67 nMOF

Seen from TEM images, as-synthesized UiO-67 was obtained with a diameter of 70–90 nm (Fig. 1B), and the size and morphology did not have significant changes post cobalt and ruthenium incorporation. X-ray diffraction (XRD) pattern of the UiO-67 was consistent with previous reports (Fig. 1C) [32]. Also, the incorporation of different cargos (such as cobalt, ruthenium and TPZ) along with PEGylation from Py-PAA-PEG did not alter the crystalline structure of UiO-67, confirming its physical stability. PEGylation resulted in the slight elevation of material size, which could be observed from scanning electron microscopy (SEM) images (Additional file 1: Figure S1) and dynamic laser scattering (DLS) measurements (Additional file 1: Figure S2). XPS measurement indicated the successful accommodation of cobalt and ruthenium inside UiO-67 nMOF structure (Fig. 1D). The existence of cobalt inside UiO-67 was also validated by UV–Vis spectra of cobalt (Additional file 1: Figure S3). Since ruthenium could be stably integrated in UiO-67 (validated in XPS spectra from Fig. 1D), it could serve as a photosensitizer to enable the photocatalytic CO production in the following studies, which was consistent with a previous report by Gao et al. [15]. The loaded cobalt and ruthenium maintained sufficiently high stability inside UiO-67 structure, since the measurement of Co and Ru by inductively coupled plasma mass spectrometry (ICP-MS) confirmed that less than 1% metal release was found (e.g. less than 60 μg Co was released from 10 mg Co/Ru-UiO-67) at pH of 6.5 and 5.8 within the time frame of 24 h (Additional file 1: Figure S4). FT-IR revealed that the coordination of cobalt and ruthenium altered the stretching behavior of multiple chemical bonds inside UiO-67 (Additional file 1: Figure S5), while successful PEGylation could also be seen on the FT-IR spectra. Synthesized UiO-67 nMOF had very abundant porosity. Judging from nitrogen adsorption–desorption isotherms (Fig. 1E), UiO-67 had a specific surface area of 1210.3 m^2^/g, which revealed its suitability for cargo accommodation. After the loading of different cargos (cobalt, ruthenium, and TPZ), the specific surface area changed to 162.3 m^2^/g, again validating that all the cargos were actually on board. Also, based on the thermogravimetric analysis (TGA) (Additional file 1: Figure S6), Py-PAA-PEG occupied ~ 17% of the total mass in TPZ-Co/Ru-UiO-67@Py-PAA-PEG.

The CO transformation properties of Co/Ru-UiO-67 was subsequently investigated. When irradiated by an 8 W LED light at a distance of 5 cm, the CO production could be clearly observed (witnessed as the continuous air bubble formation) in the DMF Co/Ru-UiO-67 suspension with saturated CO_2_ inside (Additional file 2: Video S1), while UiO-67 without Co and Ru did not cause any observable CO formation (Additional file 3: Video S2). To quantify the CO formation rate, we used myoglobin (Mb) since it was proved as a good target system to capture CO [33]. A relationship of absorbance changes from deoxy-Mb after CO incubation was shown in Additional file 1: Figure S7. Fluorescent probe for CO (COP-1) [34] was also used to quantify the production of CO from Co/Ru-UiO-67 post laser irradiation at 473 nm and 0.25 W/cm^2^ for 10 min. Based on the standard curves from Mb and COP-1, a catalysis speed of 5.6 nmol/min per 1 mg Co/Ru-UiO-67 was calculated. All these data confirmed a CO-catalysis nanoplatform (Co/Ru-UiO-67) was developed with satisfactory catalytic efficacy.

### The interactions between Co/Ru-UiO-67 conjugates and cells

**Fig. 2:**
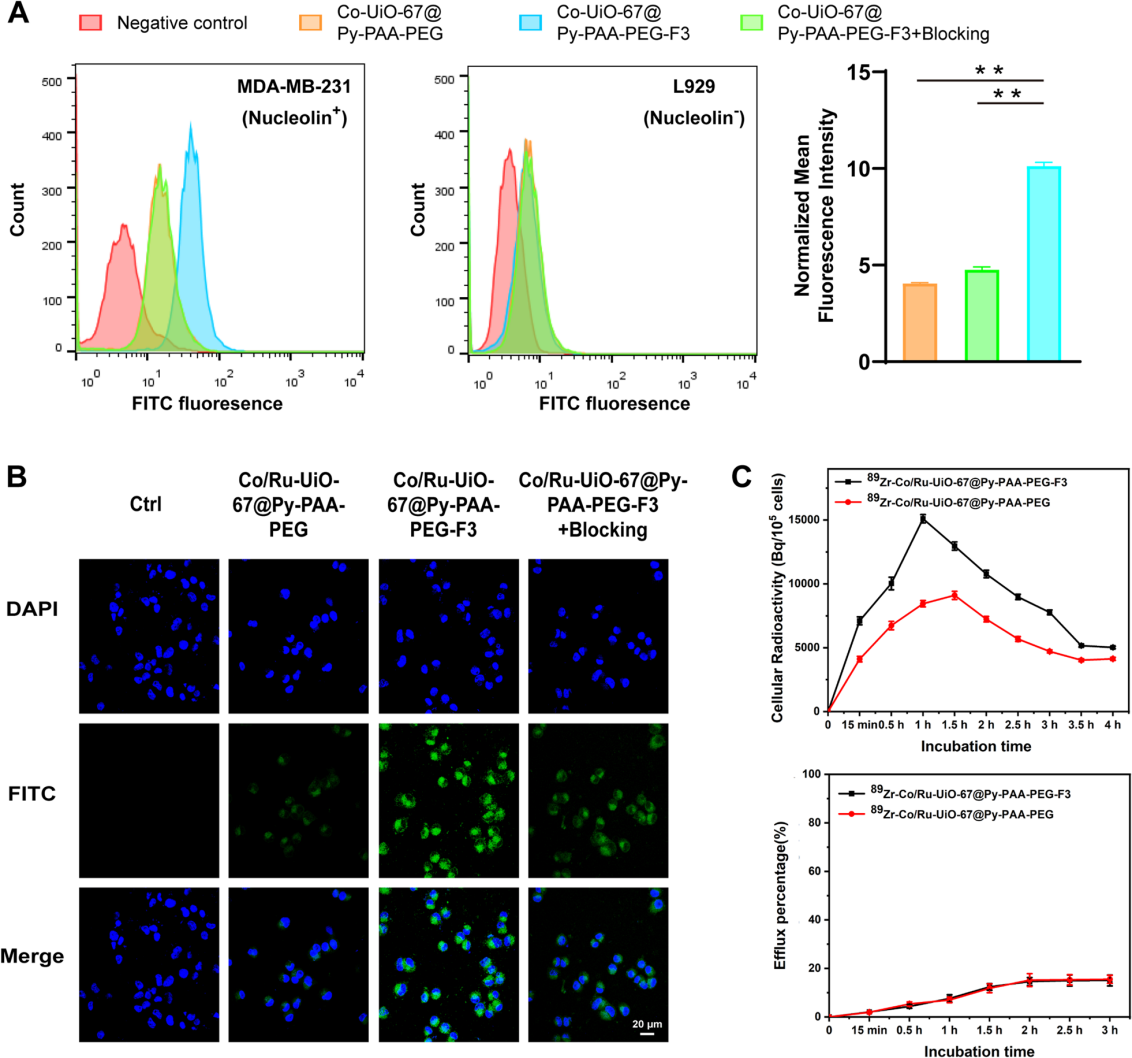
Evaluation of the interactions between UiO-67 conjugates and different cells. **A** Flow cytometry histogram results of fluorescein-labelled UiO-67 conjugates (Co-UiO-67@Py-PAA-PEG-F3 and Co-UiO-67@Py-PAA-PEG) in nucleolin-positive MDA-MB-231 cells and nucleolin-negative L929 cells. Quantification analysis of normalized mean fluorescence intensity in MDA-MB-231 cells was also shown to confirm the controlling role of F3 conjugation. **B** The confocal fluorescence microscopy results of fluorescein-labelled UiO-67 conjugates (Co/Ru-UiO-67@Py-PAA-PEG-F3 and Co/Ru-UiO-67@Py-PAA-PEG) in MDA-MB-231 cells (Blue: DAPI, Green: fluorescein-labelled UiO-67 conjugates). **C** The kinetic evaluation of cell internalization (upper) and efflux (below) behavior of ^89^Zr-Co/Ru-UiO-67@Py-PAA-PEG-F3 and ^89^Zr-Co/Ru-UiO-67@Py-PAA-PEG in MDA-MB-231 cells

After the PEGylation with Py-PAA-PEG and tumor-targeting F3 peptide, the material interactions with cancer cells (i.e. triple-negative breast cancer cells MDA-MB-231) and control cells (murine fibroblasts L929) were investigated. Learned from the flow cytometry results (Fig. 2A) and confocal fluorescence microscopy results (Fig. 2B), it was found that with PEGylation, both Co-UiO-67 and Co/Ru-UiO-67 could enter MDA-MB-231 cells, while F3 conjugation here served as the controlling factor for these UiO-67 conjugates to have the elevated entrance into MDA-MB-231 cells. As also evidenced by the competitive blocking via F3 peptide (1 μM), significantly decreased cellular uptake for Co-UiO-67@Py-PAA-PEG-F3 was seen here. In contrast, since L929 cells did not express significant amount of nucleolin for F3 peptide to bind [29], attachment of F3 peptide did not impact the material entrance into L929 cells, and UiO-67 materials’ internalization into L929 cells was discriminately lower than that in MDA-MB-231 cells.

Once zirconium-89 (^89^Zr) was incorporated into Co/Ru-UiO-67, the quantitative kinetic evaluation of material interactions with MDA-MB-231 cells was given (Fig. 2C). Based on the γ-counting measurement, the internalization of ^89^Zr-Co/Ru-UiO-67@Py-PAA-PEG-F3 reached the peak at roughly 1 h post-incubation. At almost every time point examined, the cell internalization amount of ^89^Zr-Co/Ru-UiO-67@Py-PAA-PEG-F3 was significantly higher than that of ^89^Zr-Co/Ru-UiO-67@Py-PAA-PEG, again proving the usefulness of F3 peptide conjugation. Calculated from the applied material concentration, ~ 15% of ^89^Zr-Co/Ru-UiO-67@Py-PAA-PEG-F3 could get internalized into MDA-MB-231 cells at 1 h post-incubation. On the other hand, the efflux rate of ^89^Zr-Co/Ru-UiO-67@Py-PAA-PEG-F3 was similar to that of ^89^Zr-Co/Ru-UiO-67@Py-PAA-PEG, both less than 15% at all the tested time points. These quantitative results confirmed that PEGylated and F3 conjugated Co/Ru-UiO-67 could actively enter the cancer cells, and their cellular retention was sufficiently good, which was consistent with the flow cytometry and fluorescence microscopy findings.

### The impacts of Co/Ru-UiO-67 conjugates on cancer cells

**Fig. 3:**
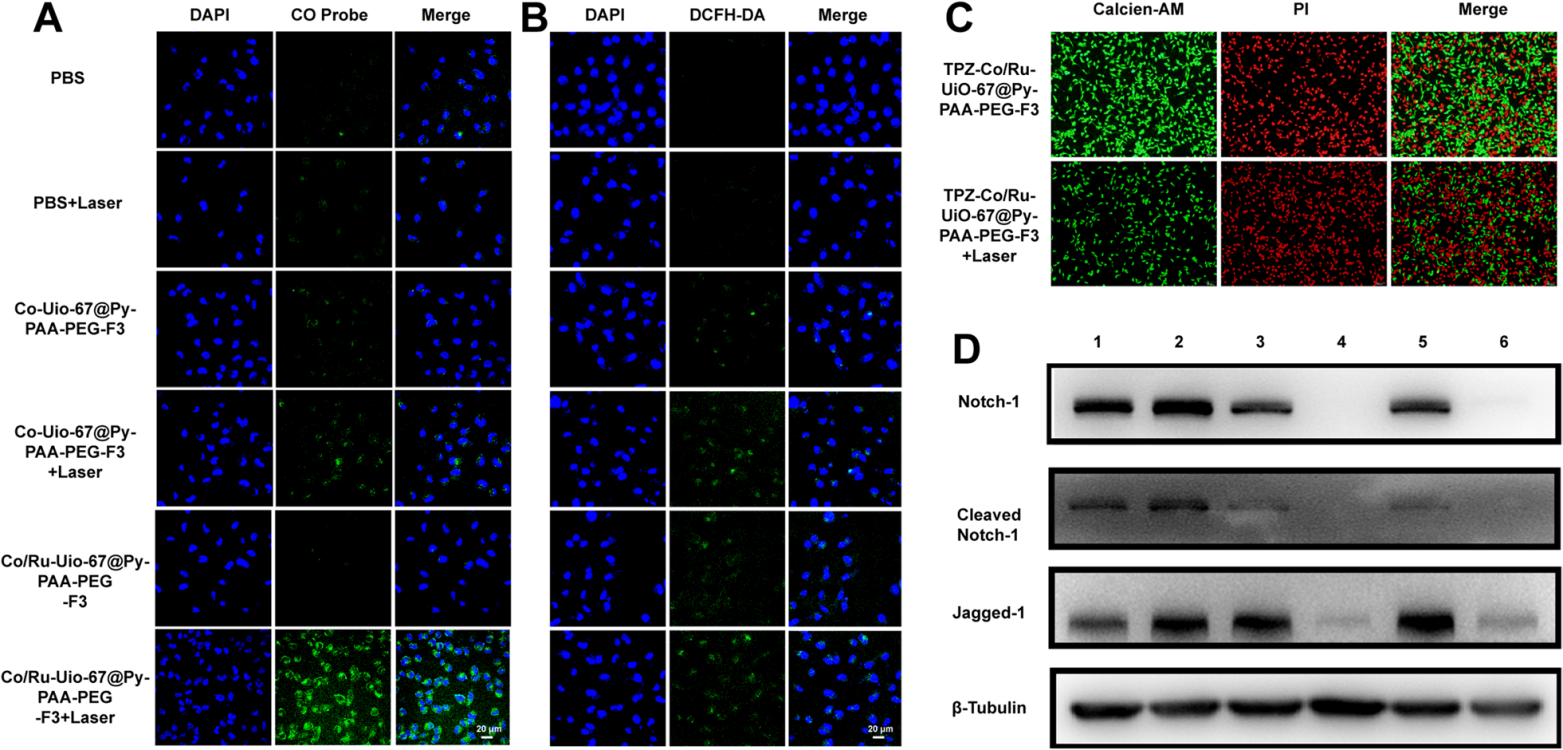
The impacts of Co/Ru-UiO-67 conjugates to cell behavior of MDA-MB-231. **A** Measurement of cellular CO by COP-1 fluorescence probe to investigate the CO catalytic efficacy by Co/Ru-UiO-67 conjugates with or without laser irradiation. **B** Measurement of cellular ROS by DCFH-DA in cells treated with Co/Ru-UiO-67 conjugates (with or without laser irradiation). **C** Calcein-AM/PI double staining to demonstrate the live/dead cells post material treatment. **D** Western blot in MDA-MB-231 cells treated with different Co/Ru-UiO-67 conjugates. 1: PBS, 2: PBS + laser, 3: Co/Ru-UiO-67@Py-PAA-PEG-F3, 4: Co/Ru-UiO-67@Py-PAA-PEG-F3 + laser, 5: Co/Ru-UiO-67@Py-PAA-PEG, 6: Co/Ru-UiO-67@Py-PAA-PEG + laser

Once the cellular internalization for Co/Ru-UiO-67 conjugates into cancer cells was confirmed, we studied the biological impact of Co/Ru-UiO-67 conjugates on MDA-MB-231 cells. As these UiO-67 conjugates entered the cells, they did not cause significant toxicity if they did not get sufficient amount of irradiation (Additional file 1: Figure S8). Firstly, we found that ruthenium incorporation efficiently increased the CO catalytic capacity of Co-UiO-67 conjugates. COP-1 probe was used in the cells to measure the CO production, which emits green fluorescence once reacts with cellular CO. Co/Ru-UiO-67@Py-PAA-PEG-F3 triggered strong cellular CO fluorescence upon the irradiation by the 473-nm laser (Fig. 3A). Co-UiO-67@Py-PAA-PEG-F3 with laser induced certain amount of CO fluorescence, however significantly lower than that of Ru-containing Co-UiO-67@Py-PAA-PEG-F3. Laser irradiation of Co/Ru-UiO-67@Py-PAA-PEG-F3 inside MDA-MB-231 cells also induced the formation of reactive oxygen species (ROS), which was witnessed by a DCFH-DA staining (Fig. 3B). In addition, laser irradiation of Co/Ru-UiO-67@Py-PAA-PEG-F3 in MDA-MB-231 cells could also influence the cellular ATP level (Additional file 1: Figure S9) and mitochondrial membrane potential (Additional file 1: Figure S10), which revealed that irradiated Co/Ru-UiO-67@Py-PAA-PEG-F3 could modify cancer cell metabolism, thus providing more selective therapeutic potentials.

Along with TPZ, irradiated Co/Ru-UiO-67@Py-PAA-PEG-F3 could induce significant amount of cell deaths in MDA-MB-231 cells (Fig. 3C), validating the synergistic effect of CO release and enhanced TPZ killing of cancer cells. Moreover, CO released from light catalysis via Co/Ru-UiO-67 platform in this report impacted the CO relevant signaling in a distinct manner. As can be clearly seen from Fig. 3D, both Co/Ru-UiO-67@Py-PAA-PEG-F3 and Co/Ru-UiO-67@Py-PAA-PEG plus laser irradiation could decrease the cellular expression of Notch-1, cleaved Notch-1, and Jagged-1. Based on some previous findings [35], CO usually activated Notch-1 to trigger the downstream biological signaling. One possible explanation to our unique findings in this study may lie in the fact that CO concentration produced in this study was different from previous reports. The Co/Ru-UiO-67 nanoplatform could trigger quite rapid transformation of CO_2_ to CO, and the large quantity of CO could cause different activation/deactivation status of relevant protein factors, such as Notch-1, Jagged-1 etc. This phenomenon caused our attention and will be studied in more details in our following research reports.

### In vivo PET imaging and organ distribution studies

**Fig. 4:**
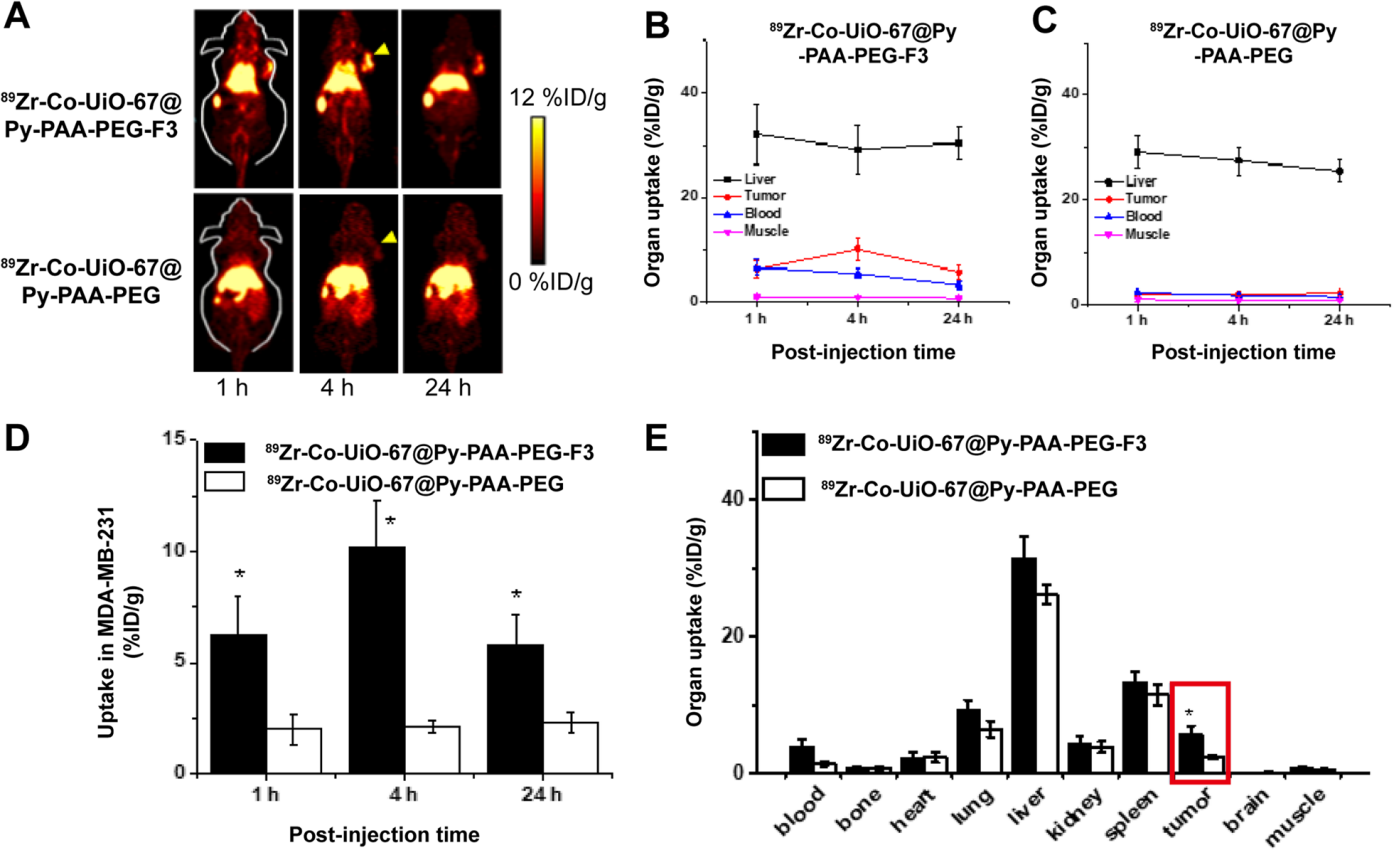
The investigation of Co-UiO-67 conjugates in vivo by PET imaging. **A** Representative PET images of MDA-MB-231 tumor bearing mice injected with ^89^Zr-Co-UiO-67@Py-PAA-PEG-F3 or ^89^Zr-Co-UiO-67@Py-PAA-PEG. **B** The region-of-interest (ROI) analysis of mice images injected with ^89^Zr-Co-UiO-67@Py-PAA-PEG-F3 (n = 4). **C** The ROI analysis of mice images injected with ^89^Zr-Co-UiO-67@Py-PAA-PEG (n = 4). **D** Tumor uptake comparison between ^89^Zr-Co-UiO-67@Py-PAA-PEG-F3 (n = 4) and ^89^Zr-Co-UiO-67@Py-PAA-PEG (n = 4). * p < 0.05. **E** Organ distribution profiles of ^89^Zr-Co-UiO-67@Py-PAA-PEG-F3 and ^89^Zr-Co-UiO-67@Py-PAA-PEG by gamma counting. Red box highlighted the tumor uptake in each group

^89^Zr-Co-UiO-67 was synthesized with a radiochemical yield of 58.6 ± 8.6% (*n* = 5), and the specificity activity was ~ 185 MBq/mg. The stability of ^89^Zr-Co-UiO-67 was quite good, since  > 99% ^89^Zr stayed on ^89^Zr-Co-UiO-67 post a 120 h incubation of mouse serum (this observation showed the PET signal in vivo could truly represent the location(s) of Co-UiO-67). After the radiolabeling and PEGylation, ^89^Zr-Co-UiO-67 was used in MDA-MB-231 tumor-bearing mice. In vivo investigation by PET imaging indicated a rapid accumulation of ^89^Zr-Co-UiO-67@Py-PAA-PEG-F3 in MDA-MB-231 tumors (observable at 1 h p.i. while peaked at 4 h with a tumor uptake of 10.2 ± 2.1% injection dose per gram [% ID/g], Additional file 1: Table S1 and Fig. 4A, B, D) while the tumor uptake of ^89^Zr-Co-UiO-67@Py-PAA-PEG was significantly lower than that of ^89^Zr-Co-UiO-67@Py-PAA-PEG-F3 at all time points examined (2.1 ± 0.3%ID/g at 4 h p.i., Additional file 1: Table S1 and Fig. 4A, C, D). These results all confirmed the strong in vivo tumor-targeting efficacy of ^89^Zr-Co-UiO-67@Py-PAA-PEG-F3 in these living animals. ^89^Zr-Co-UiO-67@Py-PAA-PEG-F3 had relatively strong and persistent uptakes in liver at all time points examined (Additional file 1: Table S1). In addition, the circulation clearance of ^89^Zr-Co-UiO-67@Py-PAA-PEG-F3 was relatively fast for a nanomaterial (circulation half-life was calculated to be ~ 0.6 h, Additional file 1: Figure S11). It was confirmed in PET imaging studies that ^89^Zr-Co-UiO-67@Py-PAA-PEG-F3 could truly serve as a good tumor-targeting platform with satisfactory tumor-to-background ratio.

Immediately after the last PET scans at 24 h p.i., biodistribution studies by gamma counting were conducted to confirm that quantitative tracer uptake values based on PET imaging accurately reflected radioactivity distribution in tumor-bearing mice, as similar %injection dose per gram (% ID/g) values were obtained from PET and biodistribution studies (Fig. 4E). Liver still had significant tracer uptake for ^89^Zr-Co-UiO-67@Py-PAA-PEG-F3, since the tracer was primarily excreted from the hepatobiliary pathway. More importantly, the accumulation of ^89^Zr-Co-UiO-67@Py-PAA-PEG-F3 in MDA-MB-231 tumor was sufficiently high and persistent, maintaining good tumor detection capacity.

### Therapeutic evaluations

**Fig. 5:**
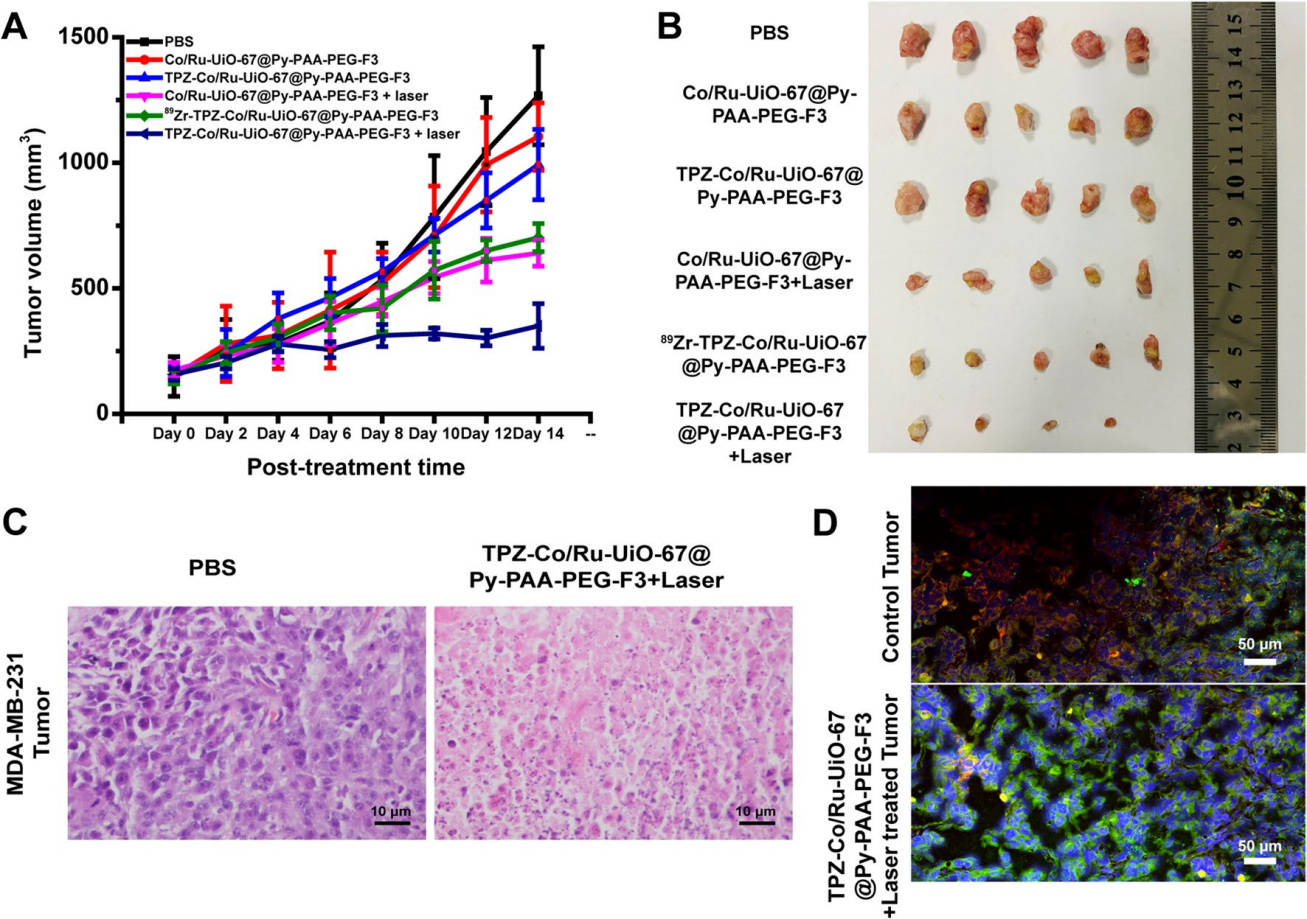
The therapeutic performance of Co/Ru-UiO-67 conjugates in vivo. **A** MDA-MB-231 tumor growth curves of different material treated groups (n = 5). **B** Photos of excised tumors of each treatment group (n = 5). **C** H&E staining of control and TPZ-Co/Ru-UiO-67@Py-PAA-PEG-F3 treated tumor (n = 5). **D** Immuofluorescence staining of control and TPZ-Co/Ru-UiO-67@Py-PAA-PEG-F3 treated tumors (Blue: DAPI, Green: CO profiles, Red: CD31 (tumor vasculature marker)) (n = 5)

Co/Ru-UiO-67 conjugates were administrated to mice via intravenous injection, and the tumor volumes were continuously monitored for 2 weeks (Fig. 5A). The dosage of all Co/Ru-UiO-67 conjugates was 10 mg/kg for each mouse. Fast-growing tumor volumes were observed in PBS, Co/Ru-UiO-67@Py-PAA-PEG-F3 and TPZ-Co/Ru-UiO-67@Py-PAA-PEG-F3 treated mice. In contrast, a modest inhibition of tumor growth was achieved in mice receiving Co/Ru-UiO-67@Py-PAA-PEG-F3 with laser irradiation, and also as shown in Fig. 5A, a 50.9% reduction of the relative tumor weight on the 14th day was observed. Notably, the most effective anti-tumor effect was realized in TPZ-Co/Ru-UiO-67@Py-PAA-PEG-F3 group with laser irradiation that tumor weight at the last day was reduced by 72.4% (tumor in one mouse completely disappeared, Fig. 5B). H&E staining analysis was applied to the isolated tumors. Massive cell apoptosis was seen in the tumor tissue treated with TPZ-Co/Ru-UiO-67@Py-PAA-PEG-F3 plus laser irradiation (Fig. 5C). In addition, ^89^Zr-TPZ-Co/Ru-UiO-67@Py-PAA-PEG-F3 had sufficiently good tumor inhibition, which was comparable to that from Co/Ru-UiO-67@Py-PAA-PEG-F3 with laser irradiation. This result confirmed that treatment of deep-tissue tumor with ^89^Zr-Co/Ru-UiO-67 conjugates could be possible without the requirement of an external light source, which might sometimes cause extra cumbersome situation in clinics. Also, after the MDA-MB-231 tumors were treated with TPZ-Co/Ru-UiO-67@Py-PAA-PEG-F3, COP-1 was injected locally into the tumor for visualization of CO. Simultaneously, tumor slices were subjected to CD31 staining. The results indicated that CO was formed during the tumor treatment, while the vasculature density was decreased post TPZ-Co/Ru-UiO-67@Py-PAA-PEG-F3 + laser treatment (Fig. 5D).

We feel that CO produced from Co/Ru-UiO-67 could be the leading reason for providing an optimal environment for TPZ to get the maximal function. In addition, we also explored the other possible reasons behind this good therapeutic outcome for Co/Ru-UiO-67 platform. One interesting finding from us was that light-activated Co/Ru-UiO-67@Py-PAA-PEG-F3 could promote the recruitment of CD8^+^ T cells to the tumor site (Additional file 1: Figure S12). This may help to turn the immunologically “cold” tumor into “hot” tumor to result in more persistent cancer killing effect. Based on the previous findings [36–38], there were still conflict observations on how CO could adjust the functions and activity of CD8^+^ T cells in different diseases (e.g. diabetes, tissue hyperplasia etc.). However, the relationship between CO and CD8^+^ T cells in cancer therapeutics should be further explored, and we hope to use the Co/Ru-UiO-67 platform as a good CO “director” to simplify the CO-immune system investigations in cancer. Meanwhile, no significant tissue morphological change or blood biochemical factor alteration was observed in all groups, indicating the safety and biocompatibility of the nanoparticles used here, which was further confirmed by H&E staining of major organs (Additional file 1: Figures S13, S14). We also investigated the safety of Co/Ru-UiO-67@Py-PAA-PEG in normal Balb/c mice. Results showed that there was no significant change of body weight in mice injected with Co/Ru-UiO-67@Py-PAA-PEG (10 mg/kg), demonstrating biocompatibility of this nanoplatform (Additional file 1: Figure S15).

## Conclusions

In this study, a cobalt and ruthenium containing UiO-67 nMOF (Co/Ru-UiO-67) was developed for “targeted” synergistic tumor treatment based on light-initiated CO catalysis. Our findings indicated that Co/Ru-UiO-67 nanoplatform could produce CO in a controllable manner both in vitro and in vivo, and the formed CO could affect the downstream signaling to enhance the treatment power of TPZ. With the proper surface modification and attachment of a tumor-targeting peptide (F3), Co/Ru-UiO-67 could selectively enter triple-negative breast cancer cells and have a long cellular retention. In vivo PET imaging investigations also demonstrated the good pharmacokinetic performance of these Co/Ru-UiO-67 conjugates. The primary benefit of this Co/Ru-UiO-67 nanoplatform lies in its ability to fine tune the tumor microenvironment. Therefore, when applied in vivo, it can work with various drug cargos (e.g. TPZ in this report) to efficiently activate the killing functions of the drugs. The combination of Co/Ru-UiO-67@Py-PAA-PEG-F3 (10 mg/kg) with TPZ showed a more than 70% tumor suppression rate in the subcutaneous MDA-MB-231 tumor model with low systemic toxicity. These findings indicated that the synergistic work between CO-release platform and activatable drugs, was really a potential therapeutic approach for TNBC treatment.

## Methods

### Materials

2,2′-Bipyridine-5,5′-dicarboxylic acid (H_2_bpydc) and 4,4′-Biphenyldicarboxylic acid (H_2_bpdc) were purchased from Chemsoon (Shanghai, China). Zirconium oxychloride octahydrate was acquired from Honeywell (Morris Plains, NJ). Polyacrylic acid (PAA) was obtained from Ruixibio (Xi’an, China). F3 peptide with cysteine at C-terminal (sequence: KDEPQRRSARLSAKPAPPKPEPKPKKAPAKKC) was synthesized by QYaoBio (Shanghai, China). Fluorescein isothiocyanate (FITC), cobalt chloride (CoCl_2_), and pyrene (Py)-amine were got from Thermo-Fisher. Antibodies against CD31, GAPDH, Jagged 1 etc. were all got from Abcam. All the other chemicals and solvents were acquired from Sigma-Aldrich and used without further purifications.

### Synthesis of UiO-67 and Co/Ru-UiO-67

UiO-67 was synthesized with a previously established method with slight modifications [39]. Briefly, zirconium oxychloride (ZrOCl_2_, 0.04 mmoL) was dissolved in a mixed solution of DMF (10 mL) and acetic acid (0.5 mL). Sonication of 30 min was carried out to form a transparent solution. Subsequently, H_2_bpydc (0.012 mmol) and H_2_bpdc (0.028 mmol) were added into the solution and the whole mixture was transferred into a 25 mL Teflon autoclave and reacted under 90 ℃ for 12 h. The resulting UiO-67 materials were washed repeatedly by DMF and ethanol (three times at least for each solvent). To acquire Co/Ru-UiO-67, 10 mg of UiO-67 was suspended in 10 mL of THF, and CoCl_2_ (0.15 mmol) along with tris(2,2′-bipyridine)ruthenium(II) hexafluorophosphate (0.1 mmol) was added into the suspension. With overnight stirring at room temperature under nitrogen gas protection, Co-UiO-67 (without Ru(II) added) and Co/Ru-UiO-67 were obtained after repeated THF, ethanol, and Millipore water washing and resuspended in water for further treatment.

### Material characterizations

The size of UiO-67 conjugates was measured by JEM-2100 transmission electronic microscopy (TEM, JOEL Ltd., Tokyo, Japan) and the dynamic laser scattering (DLS) measurement was carried out on Zetasizer Nano ZS (Malvern Panalytical, UK). UV–Vis spectra were recorded in a Perkin-Elmer Lambda 390 spectrometer. X-ray photoelectron spectroscopy (XPS) for UiO-67 was conducted on a PHI5000 VersaProbe (ULVAC-PHI, Japan). Thermogravimetric analysis (TGA) was carried out on a Pyris 1 differential scanning calorimeter (Perkin-Elmer).

### Cargo loading and surface functionalization

To load TPZ into Co-UiO-67 or Co/Ru-UiO-67, Co-UiO-67 or Co/Ru-UiO-67 (10 mg) and TPZ (5 mg) were stirred in a mixed solution of DMSO and ethanol (0.5 mL + 0.5 mL) for 12 h. Since the physical properties and physiological performance of Co-UiO-67 and Co/Ru-UiO-67 were quite similar, we used Co/Ru-UiO-67 under light catalysis condition. Other than that, Co-UiO-67 was selected as the “target” material in the relevant evaluations for clarity reasons. Centrifugation (5000 rpm* 20 min) was used to separate TPZ-loaded Co/Ru-UiO-67 (or Co-UiO-67) from free TPZ. The precipitates were washed with ethanol for three times and resuspended in Millipore water. After all the cargo loading, Co/Ru-UiO-67 or Co-UiO-67 was conjugated with pyrene-derived PEG molecules. The surface-modifying PEG (Py-PAA-PEG) was synthesized using a previously established method [40]. In the end of Py-PAA-PEG molecule, maleimide groups were incorporated to react with cysteine-containing F3 peptide, and the tumor-targeted materials were named as Co/Ru-UiO-67@Py-PAA-PEG-F3 or Co-UiO-67@Py-PAA-PEG-F3.

### Isotope incorporation into Co-UiO-67

Zirconium-89 oxalate was obtained from DC AMS Pharma (Nanjing, China), which was produced via ^89^Y(p, n)^89^Zr reaction with a specific activity of 352.2 ± 26.4 MBq/μg of Zr (n = 5). Zirconium-89 chloride was obtained from ^89^Zr-oxalate after the heat vaporization (200 ℃, 2 h) of oxalate by the addition of concentrated HCl. During the UiO-67 nMOF synthesis, 148 MBq of ^89^ZrCl_4_ was added into a 2-mL reaction mixture while the same reaction condition was maintained for UiO-67. After obtaining the ^89^Zr-incorporated UiO-67, cobalt embedding, Py-PAA-PEG-Mal functionalization and F3 conjugation was also carried out for in vitro and in vivo evaluation of ^89^Zr-Co-UiO-67 conjugates.

### Cell interaction studies

The dynamic interactions between Co/Ru-UiO-67 or Co-UiO-67 nMOFs and different types of cells were assessed in three ways. Flow cytometry and confocal fluorescence microscopy were used for non-radioactive Co/Ru-UiO-67 or Co-UiO-67 conjugates. FITC was covalently attached on the PEGylated Co/Ru-UiO-67@Py-PAA-PEG or Co-UiO-67@Py-PAA-PEG via the intermediate linker of 2-mercaptoethylamine. Since the attachment of F3 peptide was to introduce the nucleolin targeting capacity, two cell types with distinct nucleolin expression profiles (i.e. MDA-MB-231 (nucleolin^+^) and L929 (nucleolin^−^) cells) were used for the measurement of cell interactions. The material concentration of 100 μg/mL was chosen to evaluate the material-cell dynamics. Flow cytometry data collection post 1 h material incubation was conducted on a BD FACScalibur and the data were processed by the FlowJo software (vX.0.7). Confocal fluorescence microscopy was done on a Zeiss LSM 980 with Airyscan 2, taken at 200 × magnification.

In addition, the interaction of ^89^Zr-Co-UiO-67 conjugates and MDA-MB-231 cells was assessed by counting the radioactivity accumulated inside the cells [41]. The dynamic changes inside the cells were recorded to demonstrate the longitudinal cell internalization percentage. Briefly, MDA-MB-231 cells were suspended in PBS to a concentration of 2 × 10^5^ cells per milliliter. ^89^Zr-Co-UiO-67 conjugates were added to the cell suspension at the concentration of 10^5^ cpm/mL and the incubation was conducted at 37℃. At selected time points (0 to 4 h, every 30 min), a portion of cell suspension was taken and treated with 0.25 M sodium citrate (pH = 2) to remove the material binding to the cell surface. Cells were centrifuged and collected to count the radioactivity inside the cells, which was transferred to the material concentration per cell numbers. In addition, when the cellular internalization level of ^89^Zr-Co-UiO-67 conjugates reached the plateau, material-containing MDA-MB-231 cells were resuspended in PBS (still at the cell density of 2 × 10^5^ cells/mL), while a portion of the cell suspensions were taken and centrifuged, since the radioactivity in the supernatant represented the efflux percentage of the materials once they got internalized into the cells.

### Light-triggered CO production

To test the CO catalytic capacity of Co/Ru-UiO-67, an 8-W LED was used to irradiate Co-UiO-67 suspended in a CO_2_ saturated solution (the distance between the LED and the solution was 5 cm). At different time post incubation, a portion of the solution was taken from the mixture, and the CO concentration was quantified by either a UV measurement from myoglobin incubation or a fluorescence analysis from interactions with COP-1 (carbon monoxide probe 1) [34]. When Co/Ru-UiO-67 conjugates were used in cells, a 473 nm laser (5 W, CC-laser, Changchun, China) was used to irradiate Co/Ru-UiO-67 containing cells with an optical density of 0.25 W/cm^2^ and an irradiation time of 10 min.

### The measurement of CO relevant cell signaling changes

MDA-MB-231 cells were incubated with 50 μg/mL Co/Ru-UiO-67@Py-PAA-PEG and Co-UiO-67@Py-PAA-PEG-F3. At 1 h post-incubation, the cells were irradiated by the 473 nm laser at 0.25 W/cm^2^ for 10 min. Post laser irradiation, CO relevant signaling changes (e.g. Jagged 1, Notch-1 etc.) were measured by Western blot. In addition, mitochondria membrane hyperpolarization condition for treated cells was evaluated by the JC-1 probe. The cellular ROS level was measured by a dichloro-dihydro-fluorescein diacetate (DCFH-DA) kit, and the cellular ATP level was tested by a luminescent ATP detection kit (Abcam, ab113849). These all reflected the cellular changes post the formation of CO from light catalysis.

### In vivo PET imaging and organ distribution

All animal experiments were conducted following the approved protocols of Nanjing University Institutional Animal Care and Use Committee. These protocols also conform to the Guidelines for the Care and Use of Laboratory Animals published by the National Institutes of Health. Female nude mice (6–10 weeks) were purchased from Gem Pharmatech (Nanjing, China) for in vivo experiments. The mice were maintained at five per cage and kept at a specific pathogen-free (SPF) animal facility with 12 h light/dark cycles. Strict temperature and humidity control were available, along with the proper food and water supply.

PEGylated ^89^Zr-Co-UiO-67 conjugates (i.e. ^89^Zr-Co-UiO-67@Py-PAA-PEG and ^89^Zr-Co-UiO-67@Py-PAA-PEG-F3) were administrated into MDA-MB-231 tumor-bearing mice at the dosage of 3–5 MBq per mouse (n = 4) for PET imaging. The acquisition of 40 million events per time point were adopted in a Siemens Inveon rodent PET/CT scanner. The scanning was carried out at 1 h, 4 h and 24 h post-injection to evaluate the pharmacokinetic profile of ^89^Zr-Co-UiO-67 conjugates when the animals were anaesthetized by isoflurane + oxygen. The body temperature and heart-beat condition of the tested animals were also monitored in a real-time manner to avoid possible anesthesia-induced deaths. The quantification of organ radioactivity was conducted in the Inveon Research Workplace (IRW) software (v 4.2, Siemens Healthcare). Immediately after the last PET scanning, the animals were euthanized to collect the major organs for radioactivity counting using a Perkin-Elmer Wizard^2^ gamma counter. Both the PET quantification and gamma counting results were shown by the unit of % injection dose per gram (%ID/g).

### Biosafety evaluation

To test the possible side effects or toxicity from Co-UiO-67 conjugates, PEGylated Co-UiO-67 was administered into female Balb/c mice (n = 5) at a dose of 10 mg/kg body weight. The body weights of the tested mice were monitored for 7 days. At 7 days post injection, the mice were euthanized and the major organs were taken for hematoxylin and eosin (H&E) staining. In addition, mini chemistry panel test was conducted to investigate the overall health impact of Co-UiO-67 conjugates to the test animals at day 1 and day 7.

### Combinational cancer therapy studies

For cancer therapy studies, 2 × 10^6^ MDA-MB-231 cells were injected into female nude mice (6 – 10 weeks in age). And the treatment started when the tumor volume reached ~ 150 mm^3^. MDA-MB-231 tumor bearing mice were randomly divided into different groups (n = 5 per group). Since the PET imaging confirmed the good tumor-targeting efficacy of F3 peptide, PEGylated and F3 conjugated UiO-67 nMOFs were used here for combinational therapeutic purposes. All the therapeutics were administered intravenously, and the treatment groups included: PBS, Co/Ru-UiO-67@Py-PAA-PEG-F3 (10 mg/kg), TPZ-Co/Ru-UiO-67@Py-PAA-PEG-F3 (10 mg/kg), Co/Ru-UiO-67@Py-PAA-PEG-F3 (10 mg/kg) + laser irradiation, and TPZ-Co/Ru-UiO-67@Py-PAA-PEG-F3 (10 mg/kg) + laser irradiation. Therapeutic administration was performed on day 0 and day 7, and in irradiation groups, mice were irradiated with an 8-W LED light for 1 h each time (distance 5 cm) at 4 h post-injection of therapeutics (mice were anesthetized by a 1.5% isoflurane/oxygen flow during the irradiation process). Moreover, since ^89^Zr could be effectively embedded into UiO-67 structure, and ^89^Zr was known to have relatively strong Cerenkov luminescence [42], an additional treatment group was used with ^89^Zr-TPZ-Co/Ru-UiO-67@Py-PAA-PEG-F3 (10 mg/kg, ^89^Zr radioactivity: 11.1 MBq) to see whether Cerenkov luminescence from ^89^Zr could serve as a light trigger for CO catalysis using Co/Ru-UiO-67.

### Statistical analysis

All data were presented in the form of mean ± SD (standard deviation). GraphPad Prism 7.0 was used to analyze the data. One-way ANOVA or student’s t-test was used for the difference analysis of the experimental data. A value of P < 0.05 (*) was considered as statistically significant.

## Supplementary Information

**Additional file 1: Figure S1.** The scanning electronic microscopy (SEM) images of UiO-67 and PEGylated TPZ-loaded Co/Ru-UiO-67. **Figure S2.** The dynamic laser scattering (DLS) measurements of Co/Ru-UiO-67 and PEGylated Co/Ru-UiO-67. **Figure S3.** The UV-Vis spectra of cobalt chloride and cobalt-embedded UiO-67. **Figure S4.** The cumulative release of ruthenium and cobalt from Co/Ru-UiO-67 at different pH values. **Figure S5.** The FT-IR spectra of UiO-67 conjugates and PEGylated Co/Ru-UiO-67. **Figure S6.** The thermogravimetric analysis (TGA) of TPZ-Co/Ru-UiO-67 and PEGylated TPZ-Co/Ru-UiO-67. **Figure S7.** The absorbance curve of dexoy-myoglobin (Mb) before and after the incubation with CO. **Figure S8.** The cell toxicity of Co-UiO-67@Py-PAA-PEG-F3 and Co/Ru-UiO-67@Py-PAA-PEG-F3 in MDA-MB-231 cells (measured by an MTT assay in the dark). **Figure S9.** The measurement of ATP level in MDA-MB-231 cells post Co/Ru-UiO-67@Py-PAA-PEG-F3 treatment and laser irradiation. **Figure S10.** The measurement of mitochondrial membrane potential by JC-1 in MDA-MB-231 cells treated with TPZ-Co/Ru-UiO-67@Py-PAA-PEG-F3 with or without laser irradiation. **Figure S11.** The blood circulation profiles of ^89^Zr-Co-UiO-67@Py-PAA-PEG-F3 and ^89^Zr-Co-UiO-67@Py-PAA-PEG in mice. **Figure S12.** The impact of Co/Ru-UiO-67@Py-PAA-PEG-F3 to the infiltration of CD8^+^ T cells to the tumor site. Blue: DAPI. Green: CD8^+^ T cells. Length bar: 50 μm. **Figure S13.** The H&E staining of representative tissue slices before and after the 10 mg/kg treatment of Co/Ru-UiO-67@Py-PAA-PEG-F3. **Figure S14.** The blood chemical measurement of normal mice and mice at 1 day and 1 week post the treatment with Co/Ru-UiO-67@Py-PAA-PEG-F3 (10 mg/kg). **Figure S15.** Body weight curve of normal mice treated with Co/Ru-UiO-67@Py-PAA-PEG and PBS intravenously. **Table S1.** The region-of-interest (ROI) analysis of major organs injected with ^89^Zr-Co-UiO-67@Py-PAA-PEG-F3 and ^89^Zr-Co-UiO-67@Py-PAA-PEG in PET images (n = 4).

**Additional file 2. Video S1**. CO catalysis condition post light irradiation of Co/Ru-UiO-67 with saturated CO_2_ in DMF.

**Additional file 3. Video S2**. CO catalysis condition post light irradiation of UiO-67 with saturated CO_2_ in DMF.

## Data Availability

All data and materials are available in the manuscript.
