# Supplementary material for: A carbon monoxide releasing metal organic framework nanoplatform for synergistic treatment of triple-negative breast tumors
Source: J Nanobiotechnology. 2022 Nov 24;20:494. doi: 10.1186/s12951-022-01704-2 (PMC9685850; doi:10.1186/s12951-022-01704-2)
Supplement: Supplementary file 1 — Additional file 1: Figure S1. The scanning electronic microscopy (SEM) images of UiO-67 and PEGylated TPZ-loaded Co/Ru-UiO-67. Figure S2. The dynamic laser scattering (DLS) measurements of Co/Ru-UiO-67 and PEGylated Co/Ru-UiO-67. Figure S3. The UV-Vis spectra of cobalt chloride and cobalt-embedded UiO-67. Figure S4. The cumulative release of ruthenium and cobalt from Co/Ru-UiO-67 at different pH values. Figure S5. The FT-IR spectra of UiO-67 conjugates and PEGylated Co/Ru-UiO-67. Figure S6. The thermogravimetric analysis (TGA) of TPZ-Co/Ru-UiO-67 and PEGylated TPZ-Co/Ru-UiO-67. Figure S7. The absorbance curve of dexoy-myoglobin (Mb) before and after the incubation with CO. Figure S8. The cell toxicity of Co-UiO-67@Py-PAA-PEG-F3 and Co/Ru-UiO-67@Py-PAA-PEG-F3 in MDA-MB-231 cells (measured by an MTT assay in the dark). Figure S9. The measurement of ATP level in MDA-MB-231 cells post Co/Ru-UiO-67@Py-PAA-PEG-F3 treatment and laser irradiation. Figure S10. The measurement of mitochondrial membrane potential by JC-1 in MDA-MB-231 cells treated with TPZ-Co/Ru-UiO-67@Py-PAA-PEG-F3 with or without laser irradiation. Figure S11. The blood circulation profiles of 89Zr-Co-UiO-67@Py-PAA-PEG-F3 and 89Zr-Co-UiO-67@Py-PAA-PEG in mice. Figure S12. The impact of Co/Ru-UiO-67@Py-PAA-PEG-F3 to the infiltration of CD8+ T cells to the tumor site. Blue: DAPI. Green: CD8+ T cells. Length bar: 50 μm. Figure S13. The H&E staining of representative tissue slices before and after the 10 mg/kg treatment of Co/Ru-UiO-67@Py-PAA-PEG-F3. Figure S14. The blood chemical measurement of normal mice and mice at 1 day and 1 week post the treatment with Co/Ru-UiO-67@Py-PAA-PEG-F3 (10 mg/kg). Figure S15. Body weight curve of normal mice treated with Co/Ru-UiO-67@Py-PAA-PEG and PBS intravenously. Table S1. The region-of-interest (ROI) analysis of major organs injected with 89Zr-Co-UiO-67@Py-PAA-PEG-F3 and 89Zr-Co-UiO-67@Py-PAA-PEG in PET images (n = 4). [file 12951_2022_1704_MOESM1_ESM.docx]

**Supplemental Information for:**

**A Carbon Monoxide Releasing Metal Organic Framework Nanoplatform for Synergistic Treatment of Triple-Negative Breast Tumors**

Yiyang Cong^1^, Bo Sun^2^, Jianlun Hu^1^, Xiaoyang Li^3^, Yanan Wang^4^, Jingyi Zhang^4^, Dongzhi Yang^2^, Weifei Lu^4^, Zhi Ding^1,*^, Xiaofeng Wang^5^, Hao Hong^3,*^

^1^ State Key Laboratory of Pharmaceutical Biotechnology School of Life Sciences Nanjing University 163 Xianlin Avenue Nanjing 210093 China.

^2^ Jiangsu Key Laboratory of New Drug Research and Clinical Pharmacy, Xuzhou Medical University, Xuzhou, Jiangsu 221004, China.

^3^ State Key Laboratory of Pharmaceutical Biotechnology, Chemistry and Biomedicine Innovation Center (ChemBIC), Jiangsu Key Laboratory of Molecular Medicine, Medical School of Nanjing University, Nanjing 210093, China.

^4^ Key Laboratory of animal growth and development of Henan Province, Key Laboratory of Animal Biochemistry and Nutrition, Ministry of Agriculture and Rural Affairs, P.R. China, College of Animal Sciences and Veterinary Medicine, Henan Agriculture University, Zhengzhou, Henan 450002, China

^5^ Nanjing Key Laboratory of Advanced Functional Materials, Nanjing Xiaozhuang University, Nanjing 211171, P. R. China

Corresponding Authors:

***** (D.Y.) Tel: +86-516-83262138, E-email: [dongzhiy@xzhmu.edu.cn](mailto:dongzhiy@xzhmu.edu.cn)

(Z.D.) Tel: +86-13598083268, E-mail: dingzhi@nju.edu.cn

(H.H.) Tel: +86-13451926381; E-mail: [haohong@nju.edu.cn](mailto:haohong@nju.edu.cn)

**Figure S1** The scanning electronic microscopy (SEM) images of UiO-67 and PEGylated TPZ-loaded Co/Ru-UiO-67.


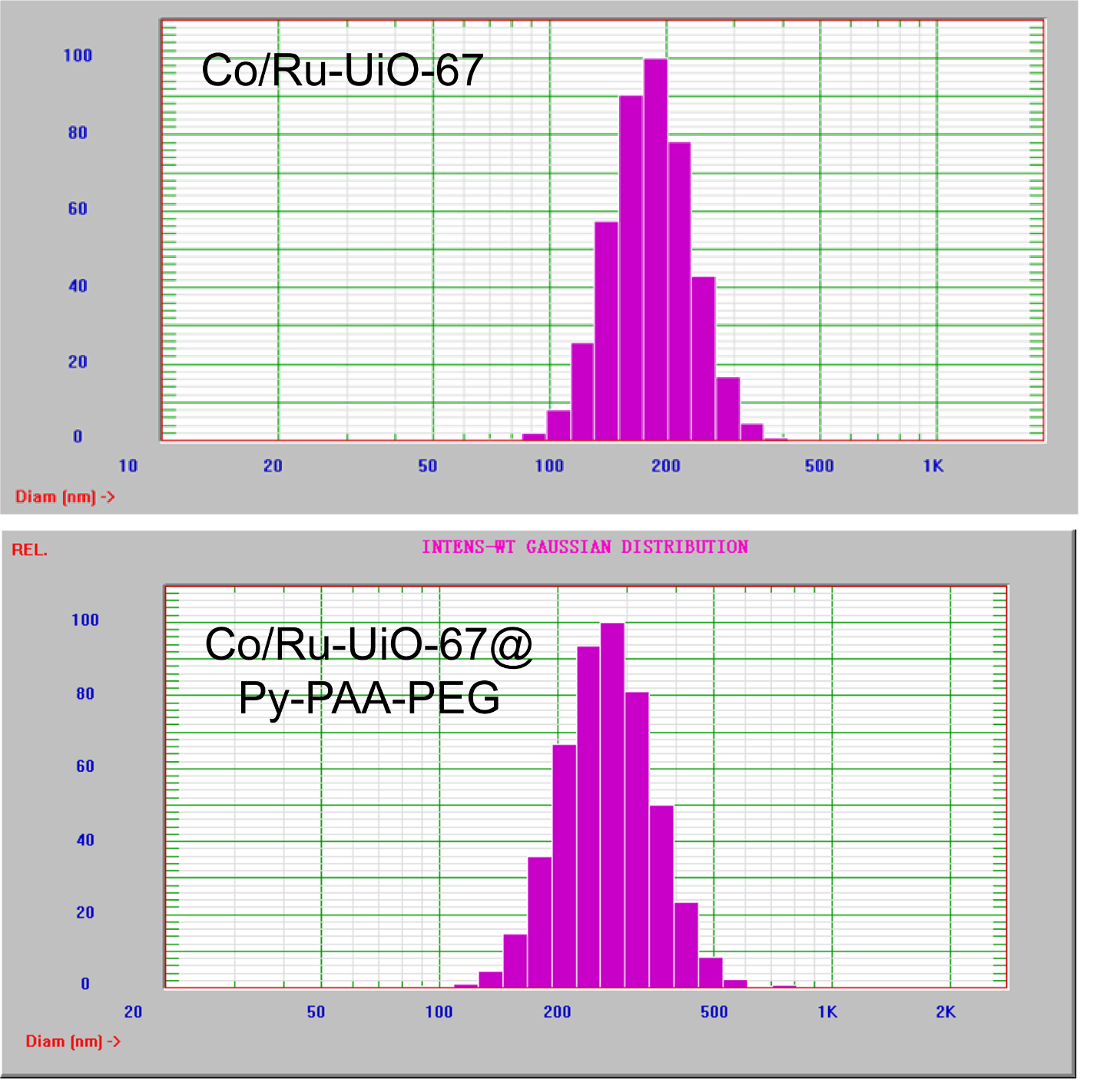


**Figure S2** The dynamic laser scattering (DLS) measurements of Co/Ru-UiO-67 and PEGylated Co/Ru-UiO-67.

**Figure S3** the UV-Vis spectra of cobalt chloride and cobalt-embedded UiO-67.

**Figure S4** the cumulative release of ruthenium and cobalt from Co/Ru-UiO-67 at different pH values.

**Figure S5** the FT-IR spectra of UiO-67 conjugates and PEGylated Co/Ru-UiO-67.

**Figure S6** The thermogravimetric analysis (TGA) analysis of TPZ-Co/Ru-UiO-67 and PEGylated TPZ-Co/Ru-UiO-67.

**Figure S7** The absorbance curve of dexoy-myoglobin (Mb) before and after the incubation of CO.

**Figure S8** The cell toxicity of Co-UiO-67@Py-PAA-PEG-F3 and Co/Ru-UiO-67@Py-PAA-PEG-F3 in MDA-MB-231 cells (measured by an MTT assay in the dark).

**Figure S9** The measurement of ATP level in MDA-MB-231 cells post Co/Ru-UiO-67@Py-PAA-PEG-F3 treatment and laser irradiation.

**Figure S10** The measurement of mitochondrial membrane potential by JC-1 in MDA-MB-231 cells treated with TPZ-Co/Ru-UiO-67@Py-PAA-PEG-F3 with or without laser irradiation.

**Figure S11** The blood circulation profiles of ^89^Zr-Co-UiO-67@Py-PAA-PEG-F3 and ^89^Zr-Co-UiO-67@Py-PAA-PEG in mice.

**Figure S12** The impact of Co/Ru-UiO-67@Py-PAA-PEG-F3 to the infiltration of CD8^+^ T cells to the tumor site. Blue: DAPI. Green: CD8^+^ T cells. Length bar: 50 μm.

**Figure S13** The H&E staining of representative tissue slices before and after the 10 mg/kg treatment of Co/Ru-UiO-67@Py-PAA-PEG-F3.

**Figure S14** The blood chemical measurement of normal mice and mice at 1 day and 1 week post the treatment with Co/Ru-UiO-67@Py-PAA-PEG-F3 (10 mg/kg).


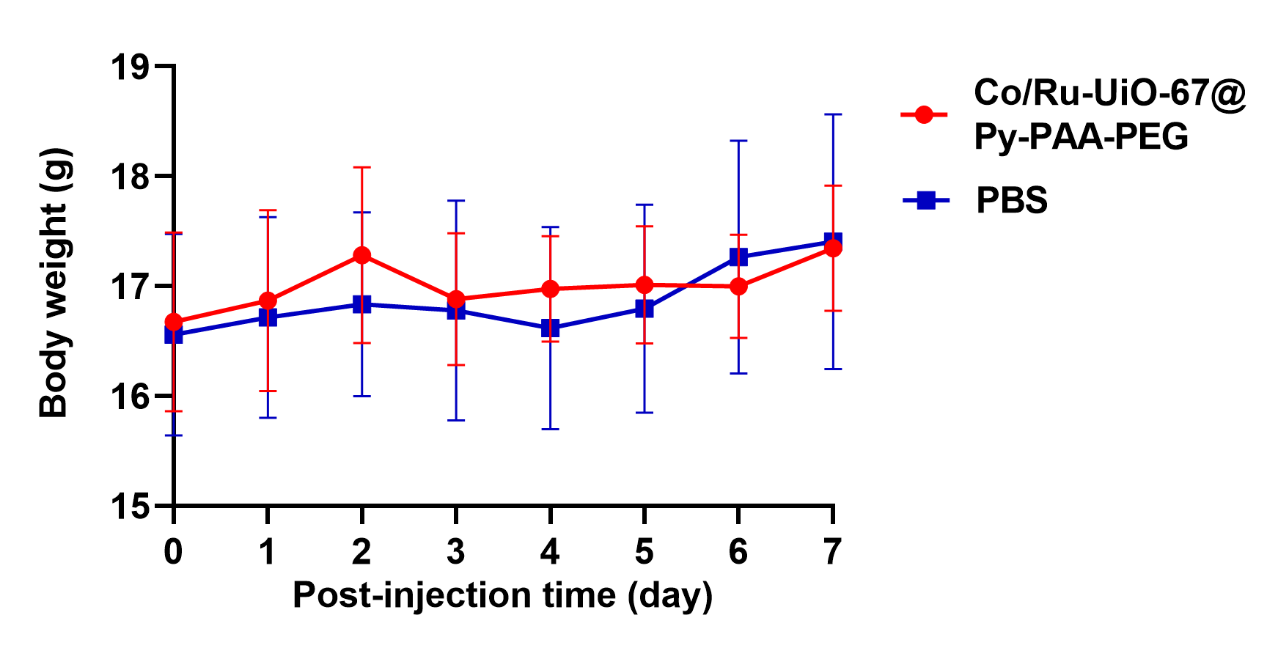


**Figure S15** Body weight curve of normal mice treated with Co/Ru-UiO-67@Py-PAA-PEG and PBS intravenously.

**Table S1** The region-of-interest (ROI) analysis of major organs injected with ^89^Zr-Co-UiO-67@Py-PAA-PEG-F3 and ^89^Zr-Co-UiO-67@Py-PAA-PEG in PET images (n = 4).

|  | Tumor (%ID/g) | Liver (%ID/g) | Blood (%ID/g) | Muscle (%ID/g) | Tumor-to-blood |
| --- | --- | --- | --- | --- | --- |
| 1 h p.i. |  |  |  |  |  |
| ^89^Zr-Co-UiO-67@Py-PAA-PEG-F3 | 6.3 ± 1.7 | 32.1 ± 5.9 | 6.7 ± 1.6 | 1.0 ± 0.4 | 0.9 ± 0.3 |
| ^89^Zr-Co-UiO-67@Py-PAA-PEG | 2.1 ± 0.4 | 30.4 ± 2.4 | 3.1 ± 0.8 | 1.0 ± 0.2 | 0.6 ± 0.2 |
| 4 h p.i. |  |  |  |  |  |
| ^89^Zr-Co-UiO-67@Py-PAA-PEG-F3 | 10.1 ± 2.1 | 29.2 ± 4.6 | 5.5 ± 0.8 | 0.9 ± 0.2 | 1.8 ± 0.4 |
| ^89^Zr-Co-UiO-67@Py-PAA-PEG | 2.2 ± 0.3 | 27.8 ± 2.5 | 2.7 ± 0.6 | 0.8 ± 0.1 | 0.7 ± 0.4 |
| 24 h p.i. |  |  |  |  |  |
| ^89^Zr-Co-UiO-67@Py-PAA-PEG-F3 | 5.8 ± 1.4 | 30.4 ± 3.2 | 3.2 ± 0.7 | 0.8 ± 0.1 | 1.9 ± 0.3 |
| ^89^Zr-Co-UiO-67@Py-PAA-PEG-F3 | 2.3 ± 0.3 | 24.5 ± 1.7 | 2.4 ± 0.5 | 0.8 ± 0.1 | 0.8 ± 0.3 |
